# Poster 2009: Clinical substantiation of immunocorrective therapy in children with atopic dermatitis

**DOI:** 10.1186/1939-4551-7-S1-P21

**Published:** 2014-02-03

**Authors:** Tatiana Slavyanskaya, Vladislava Derkach

**Affiliations:** 1 People’s Friendship University of Russia, Department of Allergology & Immunology, Moscow, Russia; 2 Pacific State Medical University, Department of Pediatrics, Vladivostok, Russia

## Background

The purpose of the study was to substantiate the need to include allergen-specific immunotherapy (ASIT) in the combined treatment (CT) of children with atopic dermatitis (AD).

## Methods

61 children aged 5 to 17 years with AD in remission who received the conventional basic therapy (BT). BT included: elimination of cause significant allergens, use of anti-inflammatory (local and systemic) therapy, correction of dysfunctions of the gastrointestinal tract. ASIT was performed during three year by an accelerated scheme for parenteral administration of the house dust mite allergens (2 injections per day with an interval of 6 hours). Children with AD were divided into 2 groups: group 1 (31 patients) against BT received under an accelerated scheme a parenteral ASIT (PASIT) mixture of house dust mite allergens. For treatment of group 2 (30 children) against BT combined immunotherapy (CIT) was used: PASIT with an immune modulator Licopid (IM) by 1 mg for 30 days, first in a dose of 10 mg, followed by 20 mg in a day exchange. The clinical efficacy of PASIT and PASIT+IM was assessed by the SCORAD index and the index of dermatological quality of life (QoL) DSQL in the time course: 1, 3, 6, 12, and 36 months after initiation of therapy. The SCORAD index in the examined groups of children before treatment was 44.71 ± 3.12 points.

## Results

When using PASIT in group 1 of the patients, the SCORAD index as well as the aggregate quality of life index DSQL after 1, 3 and 6 months of therapy remained high by the addition of a secondary infection: 39.48 ± 2.94 points (p<0.05) and 11.45 ± 0.09 points (p>0.05), respectively, had a negative impact on the children's assessment of the state of their skin. After 3 years of PASIT treatment the dermatological index QoL decreased by 2.2 times. The simultaneous use of PASIT+IM (group 2) did not provoke aggravation of AD, which enabled performing PASIT by the accelerated scheme. In neither case complications were observed. Over the 3-year course of CIT the SCORAD index truly decreased and by the end of treatment was 5.19 ± 1.11 points (p <0.001). The total index of QoL, which prior to the beginning of treatment was 11.32+0.02 points, one year after treatment it decreased 2 times, and after 36 months – 3 times (p<0.001).

## Conclusions

Inclusion of CIT in the combined treatment of children with AD has a high clinical efficacy, reduces the frequency of joining a secondary infection and improves QoL of the child.

